# Laryngeal Chondrosarcoma: an Exceptional Localisation of a not Unfrequent Bone Tumor

**DOI:** 10.1155/2009/394908

**Published:** 2009-12-29

**Authors:** Mieke Moerman, Bernard Kreps, Ramses Forsyth

**Affiliations:** ^1^Department of ENT/Head and Neck Surgery, AZ Jan Palfijn, H. Dunantlaan 5, Belgium and Maria Middelares Ghent, Kortrijksesteenweg 1025, 9000 Ghent, Belgium; ^2^Department of Histopathology, University Hospital Ghent, De Pintelaan 185, 9000 Ghent, Belgium

## Abstract

After osteosarcoma, chondrosarcoma is the second most common primary bone tumor accounting for 26% of all malignancies. In the laryngeal region however, chondrosarcomas are rather rare. Only 300 cases are reported in literature. Considering laryngeal chondrosarcoma, about 75% occur in the cricoid cartilage, whereas 20% occur in the thyroid cartilage. In this paper we report a case of thyroidal chondrosarcoma, and based on a thorough literature search we suggest some practical guidelines concerning diagnosis and therapy.

## 1. Introduction

After osteosarcoma, chondrosarcoma is the second most common primary bone tumor accounting for 26% of all malignancies [1]. In the head and neck region, chondrosarcomas most frequently occur in the maxillary bone. It is the most frequent nonepithelial tumor in the laryngeal region, accounting for 0.07–2% of all laryngeal cancers [2–6]. 

The exact cause of chondrosarcoma still remains unclear. The most common hypothesis is a primary disordered ossification of the cartilages [3]. Other theories presume a status of chronic inflammation or ischemic changes in a preexisting chondroma [2, 3, 7]. Windfuhr suggests that mechanical stress might be an important causal factor since the most frequent sites (the posterior cricoid area and the infero-lateral wall of the thyroid) correspond with muscle insertions [5]. Clinical research is still going on trying to elucidate this question. 

In this paper, a case of chondrosarcoma located in the thyroid cartilage in a 61-year-old man is reported. Practical guidelines for diagnosis and therapy are suggested. 

## 2. Case Report

A 61-year-old man presented to the outdoor department with a painless and rounded swelling at the thyroid level. The swelling had developed over years. He had no complaints, such as swallowing disorders or pain. 

**Figure 1:**
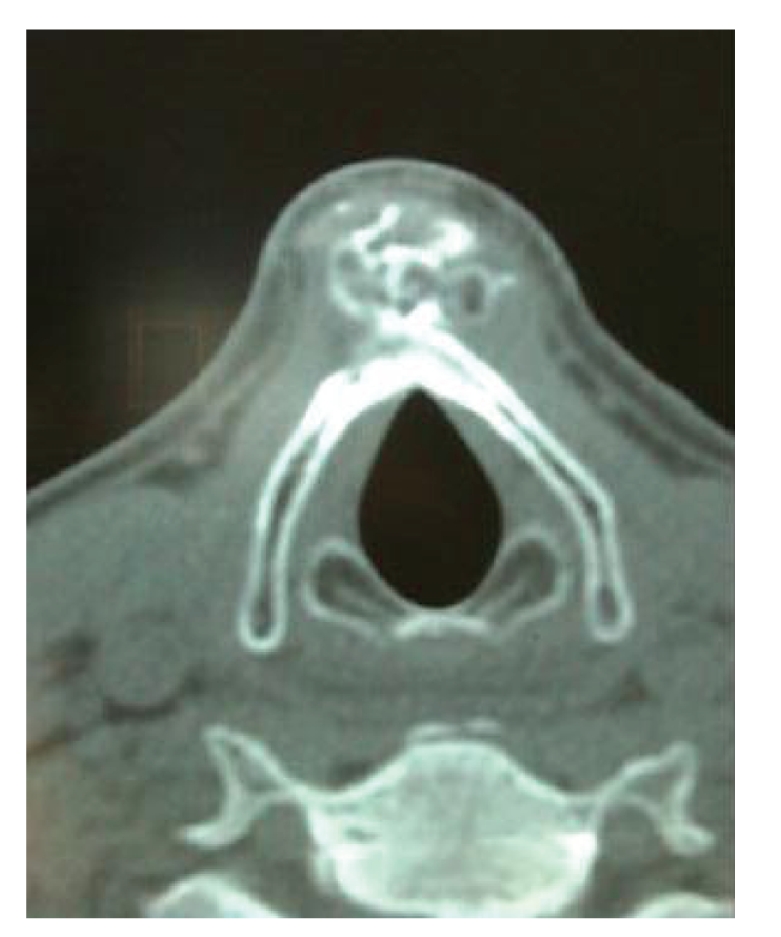
CT image of the laryngeal chondrosarcoma. This shows the lesion originating from the outer cortex with calcifications and ossifications.

Routine clinical examination revealed a tender, noninflammatory lesion at the thyroid cartilage. Endoscopy, completed with videostroboscopy and functional evaluation of swallowing (FEES), demonstrated a normal endolaryngeal and pharyngeal anatomy and physiology. Ultrasound was not conclusive but Computed Tomography (CT) provided detailed findings: a structure of 27 × 20 mm arose from the outer cortex of the thyroid cartilage anteriorly, with presentation of calcifications and ossifications (Figure 1). Pathological lymph nodes were absent. We went for a surgical exploration under general anaesthesia using an open approach through a horizontal incision in a skin crease at the level of the vocal fold's anterior commissure. Dissecting the prelaryngeal muscles cranio-caudally at the midline prompted a white firm mass. The tumor was not adherent to the soft tissues which eased the dissection. However, the median part of the outer cortex of the thyroid cartilage was clearly affected and weakened. At that moment we decided to end the operation by smoothening the outer thyroid cortex using a diamond burr, preserving the inner cortex in order not to compromise the fixation of the vocal folds (Broyle's ligament) and as a consequence causing vocal dysfunction. As it concerned a bony tumor, peroperative histologic examination (frozen sections) was not performed. 

Definite histology showed a superficially located cartilaginous tumor, largely benign in its appearance. Minimal nuclear atypia could be observed, however together with myxoid changes of the interstitium and focal invasion of the preexisting cartilage. 

Histological and clinical findings match the diagnosis of low-grade chondrosarcoma. 

## 3. Discussion

Head and neck chondrosarcomas account for 10% of all chondrosarcomas in the whole body [4]. Primary cartilaginous bone tumors of the laryngeal region are extremely rare. Laryngeal chondrosarcomas account for 0.07–2% of all laryngeal neoplasms [3–5] and less than 1% of all sarcomas [8]. It is most frequently located in the posterior lamina of the cricoid cartilage (75%), followed by the thyroid cartilage (20%), the epiglottis, and the arytenoids [2, 3]. The true incidence is difficult to assess since several low-grade chondrosarcomas have been misinterpreted as chondromas in the past [5–7]. Notwithstanding the fact that chondrosarcomas often become apparent in small foci within a vast area of chondroma tissue, even more complicating its diagnosis and thus true incidence. 

There is a male predominance, estimated between 1.29 : 1 and 4 : 1, and it occurs mostly around the sixth decade (59– 64 years) [3, 4, 6, 7, 9]. Our patient clearly fits in this profile. 

The signs and symptoms most usually observed in laryngeal chondrosarcoma are specifically: hoarseness, dysphagia, dyspnoea, and stridor. This is a consequence of impaired vocal fold mobility and/or recurrent nerve compression, eventually combined with a “mass-effect” due to endolaryngeal or exolaryngeal tumor growth. Our patient presented with a slowly growing painless swelling on the midline which did not interfere with voicing or deglutition. This suggested the origin from the outer cortex of the thyroid, confirmed by CT. In literature a delay between the onset of the tumor and the presentation of clinical findings (i.e., complaints of 28 months) is described [3]. Also this concurs with our case report. 

Plain radiology is less conclusive as it only demonstrates a variable overall density [3]. CT, however, typically demonstrates fine, punctuate, stippled to coarse “popcorn” calcifications within the tumoral mass, moderately enhancd after contrast injection [3, 5, 6]. CT also provides more detailed information concerning the site and extent of tumoral invasion [9], in this case thus providing information about the feasibility of a voice-spearing resection. In our patient the CT showed a tumor arising from the outer thyroid cortex, adjacent to the vocal fold's anterior commissure, which makes a broad resection hazardous: performing a resection of the entire median part of the thyroid causes a loosing of the anterior commissure resulting in a significant voice quality change. Alternative surgical techniques have been described, such as endoscopic debulking and CO2-laser excision for endolaryngeal lesions, but seemed not relevant in our case [3, 4]. Tracheotomy was not performed as the lesion was growing extralaryngeal and did not compromise the free airway. Of course in case of endolaryngeal growth or cricoid involvement in which surgery could hamper the airway stability, tracheotomy can provide a temporal solution. However, surgical airway reconstruction techniques such as described by Delaere et al. should be taken in concern [10]. 

Lichtenstein and Jaffe well established histopathological diagnostic criteria (Table 1) and a classification was proposed by Evans et al. (Table 2) [11, 12]. However, this counts for skeletal central chondrosarcomas. Laryngeal chondrosarcomas are not that well defined, but a high cellularity, nuclear pleomorphism, and certainly invasive growth are keys to its histological diagnosis. In this case the histopathologist finally relied on focal invasion of the preexisting cartilage to formulate the diagnosis of chondrosarcoma instead of chondroma. This illustrates the need for examining the whole specimen and is in contradistinction with other authors who state that biopsies are required [3]. According to us, biopsies may be inconclusive because (i) areas of focal invasion could be missed and (ii) firmness of the lesion sometimes makes it impossible to provide representative material in biopsies. In our case we did not perform a preoperative biopsy. 

The distinction between (laryngeal) chondroma and chondrosarcoma remains difficult as most chondrosarcomas are low-grade [2, 5, 9]. This is an additional reason to perform a complete resection, whenever possible. However, in our opinion surgery must remain conservative (as in this case: spearing the anterior vocal ligament). 

Local recurrences are quite frequent [3, 5, 6, 8, 9]. It happens in 20–60% of the cases. These recurrences can present at any time ranging from a few months to several years after the initial diagnosis and treatment. Risk factors for recurrences have not yet been well established but seem associated with an incomplete resection and tumor grade. Even in recurrent cases, conservative surgical resection is recommended such as various partial laryngectomy procedures with or without reconstruction [4, 5, 10]. We agree with this method as low- grade chondrosarcomas generally show an indolent course and do not metastasize [2, 3, 9]. 

The final outcome of laryngeal low-grade chondrosarcomas is excellent. Although one study reported a decrease in overall survival in patients over 60 years old [3], the overall life expectancy does not differ from age and gender matched groups. Death from disease and/or metastasis is very uncommon [3, 5, 7, 9, 13]. This in contradistinction to higher-graded chondrosarcomas. Especially dedifferentiated chondrosarcomas tend to metastasize at an early stage, most frequently into the lungs [2, 6, 9]. These higher-graded tumors, (i.g., myxoid or dedifferentiated chondrosarcomas) obviously require a more radical therapeutic approach. 

The role of radiotherapy still is a matter of debate. Most authors suggest that in low-grade laryngeal chondrosarcoma radiotherapy only provides an alternative treatment method when surgery is not possible [2, 3, 6, 7, 9]. The sparse reports describing the use of chemotherapy in this setting are not encouraging and thus chemotherapy is not recommended [7, 9].

## 4. Conclusion

Low-grade laryngeal chondrosarcoma is a rather uncommon tumor with an indolent course. The clinical examination should comprise exolaryngeal examination (neck palpation) and endolaryngeal examination. Laryngeal functionality (voicing and deglutition) should be investigated with naso-faryngo-laryngoscopy and/or videostroboscopy. Eventually, in case of swallowing complaints an additional functional evaluation of swallowing could be interesting. 

We would advise CT or MRI as a diagnostic tool, next to the clinical examination. It is preferential to perform a complete resection for a reliable histopathological diagnosis. As a treatment strategy, complete resection is preferable but conservative surgery is sufficient as mortality is very low. Furthermore, it limits morbidity. Follow up for years is recommended to guarantee an early detection of eventual recurrence providing even then a conservative treatment.

## Figures and Tables

**Table 1:** Chondrosarcoma criteria by Lichtenstein and Jaffe [11].

(i) The presence of many cells with plump nuclei
(ii) More than an occasional cell with two such nuclei
(iii) Giant cartilage cells with single or multiple nuclei, or containing
chromatine clumps

**Table 2:** Chondrosarcoma classification by Evans et al. [12].

Grade 1	Well-differentiated (low-grade)	Small, densely staining nuclei often with multiple nuclei within one lacune

Grade 2	Moderately differentiated (intermediate grade)	Increased cellularity, significant amount of cells having moderately sized nuclei but demonstrate a low mitotic rate of less than 2 mitoses per HPF (also includes myxoid chondrosarcoma)

Grade 3	Poorly differentiated (high-grade)	More than 2 mitoses/HPF, nuclear size generally greater than seen in grade 2 (also includes dedifferentiated chondrosarcoma)

Index: HPF: High Power Field.
